# Effect of Nurse-Led Telephone Follow ups (Tele-Nursing) on Depression, Anxiety and Stress in Hemodialysis Patients

**DOI:** 10.5539/gjhs.v8n3p168

**Published:** 2015-07-27

**Authors:** Marzieh Kargar Jahromi, Shohreh Javadpour, Leila Taheri, Farzad Poorgholami

**Affiliations:** 1 Community Health Nursing, Faculty of Nursing & Para-Medicine, Jahrom University of Medical Sciences, Jahrom, Iran; 2 Critical Care Nursing, Faculty of Nursing & Para-Medicine, Jahrom University of Medical Sciences, Jahrom, Iran; 3 Pediatric Nursing, Faculty of Nursing & Para-Medicine, Jahrom University of Medical Sciences, Jahrom, Iran; 4 Medical Surgical Nursing, Faculty of Nursing & Para-Medicine, Jahrom University of Medical Sciences, Jahrom, Iran

**Keywords:** Tele-Nursing, depression, anxiety, stress, Hemodialysis patients

## Abstract

**Introduction:**

Depressive and anxious patients on hemodialysis have a higher risk of death and hospitalizations. The aim of this study was to evaluate the effect of nurse-led telephone follow ups (tele-nursing) on depression, anxiety and stress in hemodialysis patients.

**Method & Material:**

The subjects of the study who were selected based on double blind randomized clinical trial consisted of 60 patients with advanced chronic renal disease treated with hemodialysis. The patients were placed in two groups of 30 individuals. Before the intervention, a questionnaire was completed by patients. There was no telephone follow up in the control group and the patients received only routine care in the hospital. The participants allocated to the intervention group received telephone follow-up 30 days after dialysis shift, in addition to conventional treatment. Every session lasted 30 minutes, as possible. Then the DASS scale was filled out by the patients after completion of study by two groups.

**Result:**

Significant differences were observed between the two groups in the posttest regarding the dimensions scores of DASS scale.

**Conclusion:**

The result of this trial is expected to provide new knowledge to support the effective follow-up for hemodialysis patient in order to improve their emotional and health status.

## 1. Introduction

Chronic Kidney Disease (CKD) is a silent disease that is frequently diagnosed in the advanced stages, when dialysis and renal transplantation are the only choices. A dialysis patient suffers from large burden of multiple somatic symptoms (Sajjadi, Kushyar, Vaghei, & Ismeili, 2008).

Cognitive disorders and Depression are prevalent in patients with chronic kidney disease. Elderly patients undergoing hemodialysis had higher levels of anxiety than the healthy elderly (Moser, Dracup, & Evangelista, 2010). Also anxiety decreases the quality of life of these patient and increases the length of hospital stay (Chen, Liu, Yeh, Chiang, Fu, & Hsieh, 2013).

The mean prevalence of clinical depression among hemodialysis patients approximately range from 20 to 30% with as many as in 42% of hemodialysis patients exhibiting some type of depressive affect (Hedayati, Grambow, Szczech, Stechuchak, Allen, & Bosworth, 2005; Watnick, Wang, Demadura, & Ganzini, 2005). These rates are substantially higher than the general population, where rates of depression are between 3% and 6%, and in older adults, where rates are between 6% and 10% (Rosengren, Hawken, & Ounpuu, 2004). Recent estimates project that, by 2050, rates of depression will increase by 35% in adults and more than double in older adults (Wassertheil-Smoller, Shumaker, & Ockene, 2004).

Despite the high prevalence (up to 60%) and destructive consequences, depression is still a misdiagnosed disorder; It may be due to the superposed symptoms related to uremia (anorexia, fatigue, sleep disorders) and the absence of a systematic psychiatric evaluation (Bruce & Leaf, 2012).

The importance of the management of depression has been recently the center of focus in the nephrology literature. Screening of the patients for depression and providing proper care, has been recommended by several authors (Hedayati, Yalamanchili, & Finkelstein, 2012). However, the large number of undiagnosed and untreated depression in dialysis patients indicate the existence of barriers to optimal mental health care in these patients (Finkelstein, Wuerth, & Finkelstein, 2010).

Various methods have been used to reduce anxiety and its consequences (Harris & Richards, 2010). Telephone follow-up is considered a low cost and easily organized intervention, and a good way to manage symptoms and early recognition of complication, reassurance and quality aftercare; and also to exchange information and provide health education (McCorkle, Siefert, Dowd, Rohinson, & Pickett, 2007).

For this reasons, this study was conducted to evaluate the effect of nurse-led telephone follow ups (tele-nursing) on depression, anxiety and stress in hemodialysis patients in Iran.

## 2. Method & Material

### 2.1 Study Design and Sample

The subjects of the study who were selected based on double blind randomized clinical trial consisted of 60 patients with advanced chronic renal disease treated with hemodialysis. The study population was patients referring to dialysis ward at Motahhari hospital of Jahrom, Iran, from September to March 2014. Patients were recruited through convenience sampling method. Inclusion criteria included age between 18 to 65 years, not having cognitive and psychological disorders, understanding Persian language with at least primary education, reaching final stage of renal disease and being constantly under treatment, undergoing at least 6 months of treatment with hemodialysis, being under treatment of three times a week for three to four hours, no renal transplantation and immigration during intervention, and no formal training in relation to dialysis. Exclusion criteria included having a history of serious or adverse experiences in the last six months, being treated with antidepressant medications, hospitalization due to acute disease, and unwillingness to continue to participate in the study.

### 2.2 Instrument

A two-part questionnaire was used for data collection. The first part included seven questions on patients’ demographic characteristics including age, sex, marital status, employment, education level, income level, and hemodialysis frequency in a week. The second part was the Depression Anxiety and Stress Scales (DASS) that is a widely used screening tool to assess symptoms of depression, anxiety, and stress in community settings. This instrument comprises three sub-scales: (1) the Depression sub-scale which measures hopelessness, low self-esteem, and low positive affect; (2) the Anxiety scale which assesses autonomic arousal, muscle-skeletal symptoms, situational anxiety and subjective experience of anxious arousal; and (3) the Stress scale which assesses tension, agitation, and negative affect. There are two forms of the DASS, the full 42-item and the short 21-item versions. Both assess the same domains. There is evidence of the validity of the DASS for the use in both clinical and community settings.

The DASS-21 consists of 21 items, with 7 items in each scale measuring the respective current symptoms of depression, anxiety, and stress. The DASS-21 uses a four-point scale to rate the severity, which ranges from 0 (“not apply to me at all”) to 3 (“applied to me very much, or most of the time”). To obtain the total score and the scores for depression, anxiety, and stress, the individual score from the respective items were added up and multiplied by 2, as recommended by Lovibond and Lovibond (1995) who developed the tool. The range of the score of each area is from zero to 21, (Lovibond & Lovibond, 1995). Validity and reliability of this questionnaire was confirmed by Sahebi et al. (Sahebi, Asghari, & Salari, 2005).

### 2.3 Intervention

This study was approved by the Research Council and the Research Ethics committee of Jahrom University of Medical Sciences. Data collection was performed after explaining the research objectives, and obtaining written informed consent from the participants. All patients were assured of obscurity and confidentiality of their personal information, and the right to refuse participation or withdraw from the study at any time. Also the necessary permissions were sought from the hospital authorities and the hemodialysis unit. Sixty patients were finally recruited in the trial, 30 in each group, by considering a possible attrition.

The questionnaires were completed by patients before the intervention. There was no telephone follow up in the control group and the patients received only routine care in the hospital.

The participants allocated to the intervention group receive telephone follow-up 30 days after dialysis shift in addition to conventional treatment. The content of the call followed a script to ensure consistency. The telephone follow-up consultations are structured and contain the following key subjects: communication, cognition/development, breathing/circulation, nutrition, elimination, sleep, pain/perception, skin/tissue, sexuality/reproduction, activity and psychosocial/spirituality/culture. All interventions are conducted by the researcher responsible for this trial. Every session lasted 30 minute as possible. Finally the DASS scale was filled out by the patients after completion of study by two groups.

### 2.4 Data Analysis

The data was analyzed by SPSS software version 15 and also descriptive and analytic statistics including Independent-Samples T test, Chi square, Paired t-Test and analysis of variance.

## 3. Result

In total, 54 patients completed the study. Despite the attempt of researchers to prevent attritions of samples through attending in the field and telephone follow up, but some of the patients did not complete the study. During the research, three patients in the control group and three patients in the intervention group (one patient because of death, two due to major complications, one patient due to inaccessibility by the researcher, and two patients because of declining to do hemodialysis) were excluded from the study.

Table 1 demonstrated socio-demographic characteristics of the patients. The mean age of participants was 69.13 (SD=11.82); Chi-square test showed that both groups were similar in terms of socio demographic characteristics.

**Table 1:** Frequency distribution of the study units based on demographic variables in two groups

Group		Intervention	Control	P- value

		Relative frequency	Relative frequency	
**Sex**	Female	56	40%	0.42
	Male	44%	60%	
**Marital status**	Single	28%	16%	0.27
	Married	72%	84%	
**Employment**	Yes	40%	56%	0.42
	No	60%	44%	
**Education level**	Primary	4%	4%	0.63
	Junior high school	36%	36%	
	High school	56%	48%	
	Academic	4%	12%	
**Hemodialysis frequency in a week**	Twice	44%	28%	0.17
	Three times	56%	72%	
**Income level**	Poor (< 250$)	36%	20%	0.32
	Average (250-500$)	44%	72%	
	Good (>500$)	20%	8%	

*Note.* significance level of Chi-square test considered by P<05.

The mean of depression, anxiety and stress scores in hemodialysis patients before intervention in each of the two groups are shown in Table 2. Using independent sample t-test, no significant difference was observed between the two groups in three dimensions of DASS scale before intervention. Significant differences were observed between the two groups in the posttest regarding the dimensions scores of DASS scale. The mean scores of the intervention group were higher than those of the controls (Table 3).

**Table 2:** Mean of depression, anxiety and stress scores before the intervention in the two groups

Group Variable	Intervention		Control		P-value

	M	SD	M	SD	
**Depression Score**	16.60	1.50	16.72	1.83	P=0.4
**Anxiety Score**	16.48	1.85	16.78	1.87	P=0.5
**Stress Score**	16.92	0.90	15.92	1.44	P=0.4

*Note.* significance level considered by P<05.

**Table 3:** Mean of depression, anxiety and stress scores after the intervention in the two groups

Group Variable	Intervention		Control		P-value

	M	SD	M	SD	
**Depression Score**	8.96	1.17	16.20	1.60	P=0.05
**Anxiety Score**	8.68	0.90	16.72	1.98	P=0.01
**Stress Score**	8.36	1.03	13.76	1.44	P=0.001

*Note.* significance level considered by P<05.

## 4. Discussion

This research was conducted to evaluate the effect of nurse-led telephone follow ups (tele-nursing) on depression, anxiety and stress in hemodialysis patients. Since the prevalence of chronic kidney disease is high in developing countries like Iran, we assess this population. Based on our findings, significant difference between the two groups regarding the dimensions scores of DASS scale after intervention. We found that tele-nursing program was associated with lower depression, anxiety and stress in intervention versus control group.

In a recent analysis, it was demonstrated that hemodialysis patients on dialysis had a higher risk of depression and anxiety. In other word anxiety and depression are the common non-psychotic mental disorders experienced by hemodialysis patients (Duarte, Miyazaki, Blay, & Sesso, 2009).

The effectiveness of non-pharmacologic interventions in reducing anxiety and depression has been proven in a few other reports as well. In one recent randomized controlled trial, cognitive behavioral therapy for 3 months improved depressive symptom scores significantly (Magela Salomé, de Almeida, & Silveira, 2014). A randomized controlled trial on exercise training reported promising results (Ouzouni, Kouidi, Sioulis, Grekas, & Deligiannis, 2009). Hosseini and colleagues (2009) concluded that psychological training is an appropriate alternative for physician when exposing depressed patients with kidney failure (Hosseini, Espahbodi, Mirzadeh Goudarzi, 2012).

Sundsli et al. (2014) in an article titled “self-care telephone talks as a health-promotion intervention in urban home-living persons 75+ years of age”, concluded self-care telephone talks significantly improved mental health (Sundsli, Soderhamn, Espnes, & Soderhamn, 2014).

Rollman and colleagues (2009) found that telephone follow-up is associated with clinically significant improvements in health-related quality of life (Rollman, 2009).

Kirsten et al. (2014) in their study titled “telephone follow-up by nurse following total knee arthroplasty” have shown care related to information and contact with health professionals by telephone follow-up positively improved patient satisfaction (Kirsten, Konradsen, Solgaard, & Ostergaard, 2014).

Consistent with previous findings, in our study the telephone follow-up consultations containing the following key subjects: communication, cognition/development, breathing/circulation, nutrition, elimination, sleep, pain/perception, skin/tissue, sexuality/reproduction, activity and psychosocial/spirituality/culture improved the depressive symptom in intervention group.

Based on these findings, it was considered pertinent to assess the best scientific evidence regarding to the telephone follow-up and to obtain information about working with mobile and the clinical and psychological effects for the individuals who received these interventions (Flanagan, 2009).

Tele nursing as a field, it is part of the telephone follow-up method; which mainly act as a form of monitoring for high-risk patients expose to invasive procedures, allowing them to lead a life as normal as possible. This care strategy is considered better for the service, by reducing the workload in the conventional outpatient monitoring system, allowing the professionals to focus on care for the patients who actually need hospital care (Mistiaen & Poot, 2006), which also reduces the patient's risk of readmissions and Basically, enhance their psychological symptom (Thompson-Coon et al., 2013; Naffe, 2013).

## 5. Conclusion

The result of this trial is expected to provide new knowledge to support the effective follow-up for hemodialysis patient in order to improve their emotional and health status. We expect this trial to confirm the importance of support by a clinical specialist nurse in improving mental health of hemodialysis patients especially for those in remote regions.
